# Dietary fibre in hypertension and cardiovascular disease management: systematic review and meta-analyses

**DOI:** 10.1186/s12916-022-02328-x

**Published:** 2022-04-22

**Authors:** Andrew N. Reynolds, Ashley Akerman, Shiristi Kumar, Huyen Tran Diep Pham, Sean Coffey, Jim Mann

**Affiliations:** 1 Department of Medicine, University of Otago, Dunedin, New Zealand; 2 Riddet Institute, Palmerston North, New Zealand

**Keywords:** Coronary artery disease, Hypertension, Meta-analysis, Epidemiology, Medical education

## Abstract

**Background:**

Higher dietary fibre intakes are associated with a reduced risk of developing cardiovascular disease (CVD), and increasing intake has been shown to reduce blood pressure and other cardiometabolic risk factors. The extent to which dietary fibre can further reduce risk for those with CVD and treated with cardioprotective drugs has not been clearly established. We have examined the evidence for dietary fibre as adjunct therapy in those with CVD or hypertension.

**Methods:**

Ovid MEDLINE, Embase, PubMed, and CENTRAL were searched to June 2021. Prospective observational studies reporting on fibre intakes and mortality in those with pre-existing CVD and controlled trials of increasing fibre intakes on cardiometabolic risk factors in those with CVD or hypertension were eligible. Outcomes were mortality (studies) and cardiometabolic risk factors (trials). Data synthesis was with random effects and dose response. Certainty of evidence was assessed using GRADE.

**Results:**

Three prospective studies including 7469 adults with CVD, and 12 trials of 878 adults with CVD or hypertension were identified. Moderate certainty evidence indicates reduced all-cause mortality (relative risk, RR0.75 (95% confidence interval, CI 0.58–0.97)) when comparing higher with lower fibre intakes. Low certainty evidence from trials of adults with cardiovascular disease indicates increasing fibre intakes reduced total (mean difference, MD − 0.42 mmol/L (95%CI − 0.78 to − 0.05) and low-density lipoprotein (LDL) cholesterol (MD − 0.47mmol/L (95%CI − 0.85 to − 0.10)). High certainty evidence from trials of adults with hypertension indicates increasing fibre intakes reduces systolic (MD 4.3 mmHg (95% CI 2.2 to 5.8)) and diastolic blood pressure (MD 3.1 mmHg (95% CI 1.7 to 4.4)). Moderate and low certainty evidence indicated improvements in fasting blood glucose (MD 0.48 mmol/L (− 0.91 to − 0.05)) and LDL cholesterol (MD 0.29 mmol/L (95% CI 0.17 to 0.40)). Benefits were observed irrespective of cardioprotective drug use.

**Conclusions:**

These findings emphasise the likely benefits of promoting greater dietary fibre intakes for patients with CVD and hypertension. Further trials and cohort analyses in this area would increase confidence in these results.

**Supplementary Information:**

The online version contains supplementary material available at 10.1186/s12916-022-02328-x.

## Background

Cardiovascular disease (CVD) is the leading global cause of morbidity and mortality [1]. Insufficient intake of foods high in dietary fibre has been identified as one of the leading dietary risk factors that contribute to the burden of non-communicable diseases [2]. Our systematic review and meta-analyses [3] provide convincing evidence from prospective cohort studies and clinical trials that a high dietary fibre intake can reduce cardiometabolic events and premature mortality in generally healthy populations. We have identified comparable benefits in the management of adults with type 1 and type 2 diabetes [4]. Active pharmacological management of cardiometabolic risk in patients with established cardiovascular disease has reduced the risk of further cardiovascular events and improved survival [5]. The extent to which dietary fibre can further reduce risk for those with CVD and treated with cardioprotective drugs following an acute event has not been clearly established.

We have addressed this gap in the literature by conducting a systematic review and meta-analysis of the available data. We have identified prospective observational studies reporting on fibre intakes in those with pre-existing CVD and trials in which the effects of increasing fibre on cardiovascular risk factors have been examined in people with established CVD. We have also considered trials in which the effects of dietary fibre have been examined in hypertensive individuals because they are a readily identifiable group at high risk of developing CVD [6]. As many of those with diagnosed CVD or hypertension are likely to be treated on cardioprotective medications, this research is intended to determine the extent to which dietary fibre is a useful adjunct to the pharmacological management of this high risk group of patients.

## Methods

We followed Cochrane guidelines [7] for conducting systematic reviews, World Health Organization protocols for guideline development [8], and PRISMA reporting standards for systematic reviews and meta-analyses [9]. The protocol for this systematic review was prospectively registered CRD42018089176.

### Study eligibility

This systematic review and meta-analyses were conducted to address the question “what is the role of high fibre diets in CVD and hypertension management”. Prospective observational studies of adults with CVD that reported fibre intakes and all-cause or CVD mortality were considered eligible. Controlled trials of increasing fibre intakes in those with CVD or hypertension (SBP >130 mmHg) reporting on cardiometabolic risk factors were also identified. We included parallel and crossover trials of at least 6 weeks duration where the intervention was an increase in dietary fibre. Eligible trials included those in which participants were provided with foods or were given dietary advice relating to an increase in dietary fibre with no further advice regarding macronutrients or energy intake. Trials comparing between different types or sources of fibre were not included. Trials with additional lifestyle change, such as advice to increase physical activity, were not included.

### Literature search

We identified eligible observational studies and trials from the same online search. This strategy required publications to have a term for the patient population of interest, the dietary exposure, an outcome of interest, and the study design. The list of possible terms for the outcome was broad in order to identify a wide range of CVD outcomes and potential risk factors including both standard and exploratory measures of cardiovascular function. Details of the search procedure are provided in Additional file 1.

Ovid MEDLINE, Embase, PubMed, and the Cochrane Central Register of Controlled Trials were searched up to 10 June 2021. The online search was augmented by hand searching of reference lists to identify other potentially eligible publications. No date or language restrictions were applied to the searches. Commercially available software was used to remove duplicates and aid screening [10]. At least two reviewers independently screened all articles identified by the search strategy, with disagreements resolved through discussion with a third reviewer.

### Data extraction and risk of bias assessment

Data from eligible studies were extracted by one reviewer into an Excel spreadsheet template used in a previous review [4], with a second reviewer then checking each cell. An audit of 10% of cells selected at random was also undertaken by a third reviewer. The most adjusted values for effect size were extracted for cohort studies, while baseline and post-intervention data were extracted for controlled trials. Risk of bias in eligible studies was assessed with the Newcastle-Ottawa scale [11], trials were assessed with the Cochrane risk of bias tool [12] by two reviewers independent of each other. A description of eligible studies and trials is shown in Additional files 2, 3 and 4.

### Statistical analyses and assessment of evidence certainty

For prospective cohort studies, we considered the relationship between fibre intake and all-cause or cardiovascular disease-related mortality by comparing the highest intake quantile with the lowest intake quantile [13]. Dose response relationships were considered with restricted cubic splines in a two-stage, random effects model [14] after testing for linearity (Wald test). This process uses all available quantile values for exposure (grams of fibre per day) and outcome (relative risk of mortality). For controlled trials, we analysed the mean difference between intervention and control groups with generic inverse variance models and random effects [7]. For trials with more than one eligible intervention, the control group sample size was split accordingly to avoid unit of analysis error [7]. Additional analysis combining intervention arms before pooling multiple studies did not change the direction or significance of the results, nor reduce initial heterogeneity. Correlation coefficients were obtained from publications when reported or taken from a previous review with a larger pool of trials on increasing dietary fibre intakes [4].

We considered heterogeneity between the reported results of individual studies and trials with the I^2^ statistic [15] and the *Q* test [16], However because these are overall measures unable to provide insight on sources of heterogeneity, we applied meta-regression analyses and analyses of effect sizes standardised to the same dose to consider potential heterogeneity sources for outcomes with four or more trial arms pooled. The variables considered were dose of fibre in the intervention, intervention duration, geographical region, if placebo-controlled, if eligibility criteria included an elevated BMI, if participants were on antihypertensive medication, and if the fibre were from foods, oats, or psyllium. Small study effects, such as potential publication bias, were assessed with Egger’s test [17] and if likely, the trim and fill method to consider the direction and impact of the effect [18]. Each analysis of four or more studies was considered with an influence analysis where each study or trial was removed from the pooled estimate one at a time to consider if they substantially changed the reported result. In pools of four or more point estimates, the effect size per study was standardised to 5 g of dietary fibre per day by dividing the reported effect size by the reported daily fibre dose then multiplying by five. This process assumes linearity of association in normal population intakes of fibre and the health outcomes reported on. Analyses were performed in Stata statistical software (version 15) using the metan, metabias, metatrim, metainf, and metareg commands. After producing the pooled estimate for each outcome, we used Grading of Recommendations Assessment, Development and Evaluation (GRADE) protocols [19] to calculate absolute risk reductions from prospective observational study data, and evaluate the certainty of the body of evidence for each outcome. Full details of the analyses are shown in Additional file 5. The evidence per outcome was graded as either high, moderate, low, or very low according to the potential risk of bias and the chance that further data might change the reported results. Full GRADE tables are shown in Additional file 6.

### Patient and public involvement

It was not appropriate or possible to involve patients or the public in the design, conduct, reporting, or dissemination plans of our research.

## Results

**Fig. 1:**
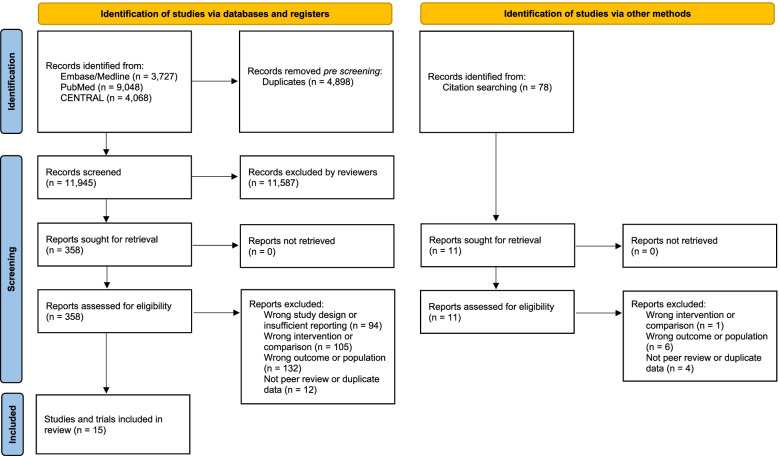
Flowchart illustrating the identification of controlled trials and prospective studies. Legend: 2020 PRISMA template of the search process undertaken to identify eligible trials and studies, with numbers of records considered or excluded at each stage of the process

The process of identifying eligible studies is shown in Fig. 1. Of the initial 16,921 titles identified, we found fifteen peer-reviewed publication that met the eligibility criteria. We identified three publications relating to four prospective observational cohort studies including 7,469 participants with CVD who were followed for a mean duration of 8.6 years. These studies were conducted in the UK [20], the USA [21], and Taiwan [22]. We also identified three eligible trials involving 230 participants with CVD [23–25], and 9 trials involving 648 participants with hypertension [26–34]. The increase in fibre intake ranged between 5.6 and 12 g per day. Trials were conducted in Asia (4), Europe (4), North America (3), and Australia (1). Eight of the trials provided fibre as supplements (tablets or powder), four trials provided oat products to increase participant fibre intake.

### Dietary fibre and premature mortality from observational studies of patients with CVD

**Fig. 2:**
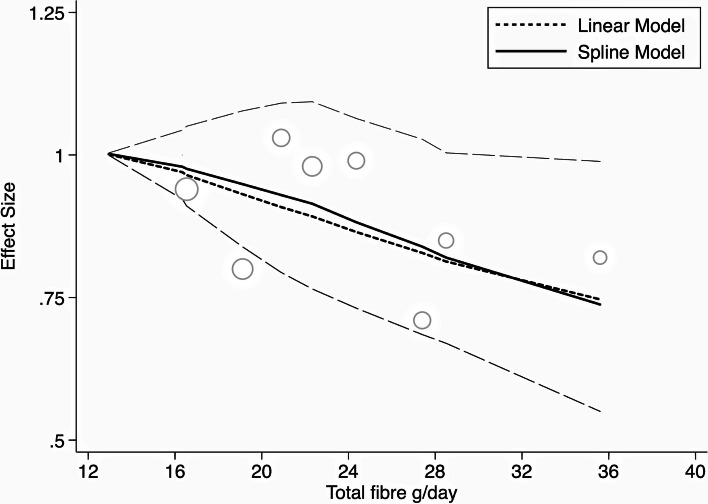
Dose-response relationship between total dietary fibre and all-cause mortality based on data from prospective studies. Legend: cubic spline and linear dose response models of total dietary fibre and relative risk of all-cause mortality in those with established heart disease. Long-dash lines are the 95% confidence intervals around the spline model risk estimate. Individual quantile data shown as circles with the larger circles having a greater influence on the model than smaller circles

Extreme quantile analyses from cohort data are shown in Table 1. A 25% reduction in all-cause mortality was observed for those consuming the most fibre when compared with those consuming the least. In terms of absolute risk, this translated into 60 fewer deaths per 1000 participants (7 to 101 fewer) for higher fibre consumers. The dose response curve for fibre and all-cause mortality is shown in Fig. 2, with an inverse relationship evident. Assuming linearity, there was a 14% risk reduction (1–26%) for every additional 10 g of fibre consumed. Risk reduction for premature mortality with higher fibre intakes was evident from data that controlled for medication use, indicating the observed benefits were independent of what is achieved in pharmacological management. The evidence for total dietary fibre intake and all-cause mortality for adults with cardiovascular disease was considered of moderate certainty following GRADE protocols. Table 1 also shows non-statistically significant decreases in mortality with higher total or cereal fibre intakes, with this body of evidence considered of very low certainty following GRADE protocols.

**Table 1:** Effects of higher compared with lower fibre intake on all-cause and cardiovascular mortality in adults with established cardiovascular disease

Exposure	Cohorts	Cases	Person years	RR (95% CI)	Absolute risk (95%CI)	Grade
**All-cause mortality**						
Dietary fibre	2	1133	41,688	0.75 (0.58 to 0.97)	60 fewer per 1000 (7 to 101 fewer)	Moderate
Cereal fibre	3	2216	62,831	0.90 (0.62 to 1.30)	33 fewer per 1000 (125 fewer to 98 more)	Very low
**Cardiovascular mortality**						
Dietary fibre	2	558	41,688	0.86 (0.60 to 1.24)	17 fewer per 1000 (47 fewer to 28 more)	Very low
Cereal fibre	4	1309	64,406	0.91 (0.64 to 1.31)	16 fewer per 1000 (63 fewer to 54 more)	Very low

### Dietary fibre and cardiometabolic risk factors in trials of patients with CVD

Meta-analyses for mean difference in cardiometabolic risk factors when increasing dietary fibre in CVD management are shown in Table 2. The available data indicate increasing fibre intakes improved measures of total and LDL cholesterol, blood pressure, blood glucose control, and body weight. Although there was high initial heterogeneity, there were insufficient data to explore with meta-regression, so the certainty of evidence for each outcome was downgraded once for Inconsistency. Further information on these analyses are shown in Additional file 5: Figs. 1-4

**Table 2:** Effects of increasing dietary fibre intakes on cardiometabolic risk factors in patients with established CVD

Outcome	Trials	Participants (I/C)	Initial ***I***^**2**^	MD (95% CI)	Grade
Total cholesterol (mmol/L)	3	117/110	99%	− 0.42 (− 0.78 to − 0.05)	Low
LDL cholesterol (mmol/L)	3	117/110	99%	− 0.47 (− 0.85 to − 0.10)	Low
HDL cholesterol (mmol/L)	3	117/110	99%	0.08 (− 0.02 to 0.17)	Low
Triglycerides (mmol/L)	3	117/110	91%	− 0.03 (− 0.15 to 0.08)	Low
Systolic blood pressure (mmHg)	1	38/38	-	− 1.2 (− 2.0 to − 0.4)	Very low
Diastolic blood pressure (mmHg)	1	38/38	-	− 3.6 (− 4.0 to − 3.2)	Very low
Body weight (kg)	1	61/53	-	− 0.20 (− 0.37 to − 0.04)	Very low
BMI (kg/m^2^)	2	99/91	98%	− 0.30 (− 0.69 to 0.09)	Low
Waist circumference (cm)	1	61/53	-	− 0.5 (− 0.6 to − 0.4)	Very low
Fasting plasma glucose (mmol/L)	2	99/91	100%	− 1.23 (− 2.13 to − 0.33)	Low
Fasting plasma insulin (pmol/L)	1	38/38	-	− 10.8 (− 13.2 to − 8.4)	Very low

### Dietary fibre and cardiometabolic risk factors in trials of patients with hypertension

The mean difference in cardiometabolic risk factors from trials of increasing dietary fibre in patients with hypertension is shown in Table 3. Results indicated improvements in systolic and diastolic blood pressure, LDL cholesterol and triglycerides and fasting plasma and insulin concentrations. The data for blood pressure improvements were consistent, with no evidence of small study effects or that other factors, including the use of anti-hypertensives, mediated the observed results. Influence analysis for both systolic and diastolic blood pressure indicated one study appreciably influenced each of the pooled results, with greater improvements seen in analyses with these studies removed (data available in Additional file 5: Figs. 15-18). Sensitivity analyses could not account for any one factor contributing to the initial heterogeneity; however, each point estimate within the meta-analyses indicated a benefit with higher fibre intake, suggesting heterogeneity was likely due to the specificity of point estimates rather than any known underlying factor. Benefits remained when standardising the fibre dose to 5 g per day, with a − 2.8 (− 3.8 to − 1.8) reduction in systolic and a − 2.1 (− 3.0 to − 1.2) reduction in diastolic blood pressure. The certainty of evidence for dietary fibre improving systolic and diastolic blood pressure was graded as high.

**Table 3:** Effects of increasing dietary fibre intakes on cardiometabolic risk factors in patients with established hypertension

Outcome	Trial arms	Participants (I/C)	Initial ***I***^**2**^	MD (95% CI)	Grade
Systolic blood pressure (mmHg)	9	281/250	99%	− 4.3 (− 5.8 to − 2.8)	High
Diastolic blood pressure (mmHg)	9	281/250	99%	− 3.1 (− 4.4 to − 1.7)	High
Total cholesterol (mmol/L)	5	190/144	98%	− 0.22 (− 0.45 to 0.01)	Low
LDL cholesterol (mmol/L)	3	137/88	97%	− 0.29 (− 0.40 to − 0.17)	Low
HDL cholesterol (mmol/L)	4	169/119	93%	0.02 (− 0.01 to 0.05)	Low
Triglycerides (mmol/L)	4	169/119	99%	− 0.19 (− 0.30 to − 0.08)	Low
Body weight (kg)	3	137/88	99%	− 0.14 (− 1.36 to 1.08)	Low
BMI (kg/m^2^)	2	92/45	99%	− 1.3 (− 2.1 to − 0.5)	Low
HbA1c (%)	1	32/31	-	0.3 (0.20 to 0.40)	Very low
Fasting plasma glucose (mmol/L)	5	195/153	99%	− 0.48 (− 0.91 to − 0.05)	Moderate
Fasting plasma insulin (pmol/L)	4	150/110	97%	− 3.5 (− 5.5 to − 1.6)	Low

There was evidence that increasing fibre intakes improved LDL cholesterol and triglyceride concentrations. Small study effects and influence analysis did not appreciably alter any pooled results. Meta-regression analyses indicated that the fibre source influenced the pooled result for total cholesterol, with greater benefits from food sources rather than supplements (− 0.52 (− 0.78 to − 0.26) *p* 0.008) with the *I*^2^ reduced to 68%. Standardising the fibre dose to 5 g per day indicated a beneficial reduction in total cholesterol (MD − 0.15 (− 0.29 to − 0.02)). The certainty of evidence for blood lipids outcomes was assessed as low following downgrading for Imprecision and Inconsistency. The data for an improvement in fasting blood glucose was assessed as Moderate following a single downgrade for inconsistency, although all sensitivity analyses indicated the finding was robust. Further information on sensitivity analyses is shown in the Additional file 5: Figs. 5-14.

## Discussion

We have considered the role of dietary fibre as a potential adjunct therapy alongside cardioprotective drugs in the management of established cardiovascular disease and hypertension. The findings indicate a reduced risk of premature mortality with higher fibre intakes when compared with lower intakes, and an improvement in key cardiometabolic risk factors when increasing fibre intakes. Risk reduction for premature mortality from the prospective observational studies was evident from data that controlled for medication use, while meta-regression from trials of adults with hypertension did not indicate anti-hypertensive medication use was a determining factor in the reported outcomes. As such, the current analyses indicate benefits with higher fibre intakes independent to what is achieved in pharmacological management.

The consideration of data from both trials of increasing fibre intakes and studies of higher intakes over time add confidence in the beneficial effects of dietary fibre intake, as the improvements in blood pressure, blood lipids, and body weight would be expected to reduce premature mortality, as was observed. There were more data available from trials of participants with hypertension than CVD, these analyses support and add to what was observed with CVD participants with improvements in blood pressure, blood lipids, bodyweight, and glycaemic control observed.

Higher dietary fibre intakes have demonstrated previous benefit in evidence synthesis on the prevention of premature mortality and non-communicable disease occurrence [3] and in diabetes management [4]. This review however is the first meta-analysis to consider the role of dietary fibre in the management of pre-existing hypertension and CVD. Furthermore, our methodology included use of meta-regression analyses to explore initial heterogeneity observed in trial data and increase confidence in the observed results. Although common, it is potentially misleading to report initial heterogeneity values without some further consideration of where it is derived. As an example, all nine data points from trials of increased dietary fibre and systolic blood pressure in patients with hypertension indicated a beneficial effect; however, the initial *I*^2^ was high (99%). Meta-regression techniques did not identify a single underlying reason for this heterogeneity, and standardisation of dose to 5g of fibre per day still produced appreciable benefits. From this we conclude that the initial heterogeneity is statistical heterogeneity due to the low variability around each point estimate, rather than underlying differences between trials beyond the interventions delivered. Our use of GRADE protocols to assess the certainty of evidence for dietary fibre intakes in these populations is a further addition to the existing literature, and a key addition for guideline development and clinical recommendations.

Current guidelines for CVD and hypertension management focus on pharmacological aides [35, 36] or if dietary, total dietary fat intake and fat quality [37]. Fewer guidelines recommend dietary fibre as part of a cardioprotective dietary pattern [38] or in lipid management [39]. The current work provides confirmation on the role of dietary fibre in human health, and the direct translatability of the findings into dietary and clinical guidelines make it a substantial contribution to the field.

Increasing dietary fibre intake led to high certainty of substantial improvements in blood pressure in adults with hypertension. These improvements were observed regardless of the use of antihypertensives. High blood pressure not only results in deleterious mechanical stress on blood vessels but also on the myocardium, leading to the development of hypertensive heart disease and congestive heart failure [1, 40]. Several pathways of action may explain this finding, such as dietary fibre’s role in reducing LDL cholesterol and triglyceride uptake [41] improving the elasticity of blood vessel walls to decrease vascular resistance and maintain adequate tissue perfusion without requiring a subsequent rise in heart rate to maintain stroke volume [42]. As a less direct mechanism, higher fibre intakes improved insulin sensitivity in this and previous works [4], with insulin sensitivity believed to play a role in endothelial dysfunction and hypertension [43]. Another major contributor to endothelial function is nitric oxide, which may be increased by increased fibre intake. Consuming foods high in dietary fibre may provide additional antioxidants [44], reducing the role of oxidative stress in the pathogenesis of atherosclerosis [45].

Other potential mechanisms for the beneficial effects observed with higher fibre intakes may relate to concomitant intakes of inorganic nitrate, or reduced body weight. High fibre foods such as vegetables also contain other beneficial nutrients that are metabolised into compounds such as nitric oxide, which may improve blood pressure through greater bioavailability for use in vasodilation [46, 47]. The current work found some support for reductions in body weight with higher fibre intakes, as shown in evidence synthesis of the general population [3] and those with diabetes [4], with weight loss beneficial in the treatment and prevention of hypertension [48]. Recent work has shown that the intake of whole grains, a considerable source of dietary fibre, when compared with refined grains leads to great measures of satiety [49], providing some rationale for why higher fibre diets may reduce energy intake through increased satiety.

The present study has many strengths, primarily the parallel consideration of the effects of increasing fibre intake from controlled trials and higher fibre intakes in prospective cohort studies enabled us to consider mechanisms supporting hard outcomes [50]. We followed recognised procedures for conducting systemic review and meta-analysis [7, 8] as well as an assessment of the certainty of evidence to support clinical and dietary guidelines [19]. To our knowledge this is the first meta-analysis to consider fibre for CVD and hypertension management, adding novelty to our work. The primary limitation of this work was the lack of relevant data available. Although only four cohort studies were identified, and it is never possible to fully exclude confounding from observational studies, follow-up duration was reasonable (weighted mean 8.6 years) and the cohorts were conducted in three distinct populations. Trials were generally of a limited number of participants, with the majority of studies of 12 weeks duration. Such limitations in the data increase the chance of observing spurious effects, although we considered that uncertainty when assessing the evidence. Further trials and cohorts of those with CVD or hypertension are needed, with some currently underway [51]. We varied from the protocol of this review by considering only trials of at least six weeks intervention duration rather than the stated two weeks. This decision was made before searches were conducted to better consider meaningful change in a broader range of cardiometabolic risk factors beyond blood lipids and blood pressure. A wider variety of interventions considering multiple food sources of fibre would increase confidence in the presented findings and may provide further evidence on the place of high dietary fibre intakes as an adjunct therapy in CVD and hypertension management.

The findings from this meta-analysis support the incorporation of high fibre foods in CVD and hypertension management, with improvement in cardiometabolic risk factors supporting the observed reduction in premature mortality. However, further trials and cohort analyses in this area would increase confidence in these results.

## Supplementary Information

**Additional file 1. **Search strategy.

**Additional file 2: Table** 1**.** Description of identified prospective studies.

**Additional file 3: Table** 1**.** Description of identified CVD trials.

**Additional file 4: Table** 1**.** Description of identified hypertension trials.

**Additional file 5: **Analyses shown in full. **Figure 1.** Fibre and all-cause mortality meta analysis. **Figure 2.** Cereal fibre and all-cause mortality meta analysis. **Figure 3.** Fibre and CVD mortality meta analysis. **Figure 4.** Cereal fibre and CVD mortality meta analysis. **Figure 5.** Fibre and total cholesterol in hypertension meta analysis. **Figure 6.** Fibre and total cholesterol in hypertension dose controlled meta analysis. **Figure 7.** Fibre and HDL cholesterol in hypertension meta analysis. **Figure 8.** Fibre and HDL cholesterol in hypertension dose controlled meta analysis. **Figure 9.** Fibre and triglycerides in hypertension meta analysis. **Figure 10.** Fibre and triglycerides in hypertension dose controlled meta analysis. **Figure 11.** Fibre and fasting plasma glucose in hypertension meta analysis. **Figure 12.** Fibre and fasting plasma glucose in hypertension dose controlled meta analysis. **Figure 13.** Fibre and fasting plasma insulin in hypertension meta analysis. **Figure 14.** Fibre and fasting plasma insulin in hypertension dose controlled meta analysis. **Figure 15.** Fibre and systolic blood pressure in hypertension meta analysis. **Figure 16.** Fibre and systolic blood pressure in hypertension dose controlled meta analysis. **Figure 17.** Fibre and diastolic blood pressure in hypertension meta analysis. **Figure 18.** Fibre and diastolic blood pressure in hypertension dose controlled meta analysis.

**Additional file 6. **GRADE tables of certainty of evidence. **Table 1.** GRADE table of fibre and mortality. **Table 2.** GRADE table of fibre in CVD management. **Table 3.** GRADE table of fibre in hypertension management.

## Data Availability

All data generated or analysed during this study are included in this published article and its supplementary information files. Effect size estimates and study details were extracted from the original papers, which are available in the public domain.

## Abbreviations

CVD: Cardiovascular disease
GRADE: Grading of Recommendations Assessment, Development and Evaluation
HDL: High-density lipoprotein
LDL: Low-density lipoprotein
MD: Mean difference
PRISMA: Preferred reporting items for systematic reviews and meta-analyses
