# Supplementary material for: Dietary fibre in hypertension and cardiovascular disease management: systematic review and meta-analyses
Source: BMC Med. 2022 Apr 22;20:139. doi: 10.1186/s12916-022-02328-x (PMC9027105; doi:10.1186/s12916-022-02328-x)
Supplement: Supplementary file 1 — Additional file 1. Search strategy. [file 12916_2022_2328_MOESM1_ESM.docx]

**Additional file 1 Search strategy applied**

**Terms for exposure**

“Dietary carbohydrate” OR “whole grain” OR wholegrain OR whole-grain OR “dietary fibre” OR “dietary fiber” OR CHO OR fruit OR vegetable OR legume OR lentil OR bean OR “pulse intake” OR “pulse consumption​”

AND

**Terms for study design**

“Randomised controlled trial” OR “randomized controlled trial” OR “parallel trial” OR “crossover trial” OR “clinical trial” OR “systematic review” OR “prospective observational study” OR “cohort study” OR “nested case-control” OR “nested case control” OR “nested cohort”

 AND

**Terms for outcome**

“Blood pressure” OR “systolic blood pressure” OR “diastolic blood pressure” OR SBP OR DBP OR “mean arterial blood pressure” OR “blood lipid profile” OR cholesterol OR LDL OR HDL OR triglycerides OR “cardiovascular function” OR “stroke volume” OR “cardiac output” OR “ankle-brachial index” OR “pulse wave velocity” OR “flow mediated dilation” OR flow “mediated dilatation” OR “coronary arterial calcium score” OR “augmentation index” OR “left ventricular mass” OR echocard OR “pulse pressure” OR “ejection fraction” OR “relative wall thickness” OR MRI

AND

**Terms for patient population**

Cardiov OR “cardiac event” OR cerebr OR CVA OR stroke OR “ischemic stroke” OR “ischaemic stroke” OR “haemorrhagic stroke” OR “hemorrhagic stroke” OR “transient ischemic attack” OR “transient ischaemic attack” OR “coronary heart disease” OR CHD OR CVD OR “heart attack” OR HBP OR hypertension OR “elevated blood pressure” OR “high blood pressure” OR “stage 1 hypertension” OR “stage 2 hypertension” OR “myocardial infarct” OR MI OR “coronary artery disease” OR CAD OR “atrial fibrillation” OR cardiomyopathy OR “heart failure” OR “ischemic heart disease” OR “ischaemic heart disease” OR “acute coronary syndrome” OR “coronary artery disease” OR atherosclerosis OR arteriosclerosis OR “apparent mineralocorticoid excess syndrome” OR “ischemic syndrome”
