# Supplementary material for: Dietary fibre in hypertension and cardiovascular disease management: systematic review and meta-analyses
Source: BMC Med. 2022 Apr 22;20:139. doi: 10.1186/s12916-022-02328-x (PMC9027105; doi:10.1186/s12916-022-02328-x)
Supplement: Supplementary file 2 — Additional file 2: Table 1. Description of identified prospective studies. [file 12916_2022_2328_MOESM2_ESM.docx]

**Additional file 2 Table 1: Description of identified prospective observational studies of adults with cardiovascular disease reporting on fibre intake and mortality.**

| ID | Cohort | Participants | Exposures of interest | Outcomes of interest | Adjustments | Newcastle Ottawa Score |
| --- | --- | --- | --- | --- | --- | --- |
| Li 2014 **USA ^1^** | Subset of the NHS and HPFS cohorts | 2639 women followed for a mean 8.7 years and 2081 men followed for a mean 9.0 years following a myocardial infarction | Dietary fibre and cereal fibre intake obtained from FFQ | 1133 all-cause deaths, 558 due to cardiovascular disease | Time since MI onset, age at diagnosis, calendar year, energy intake, physical activity, aspirin use, diabetes, high blood pressure, use of lipid lowering drugs, alcohol, saturated fat, n3 fatty acid, trans fatty acid, body mass index, coronary artery bypass surgery, folate intake, and pre-MI intake, smoking. For women, additionally adjusted for postmenopausal hormone use. For men, additionally adjusted for heart failure, left ventricular ejection fraction, and acute therapy during admission to hospital. | Exposed cohort representative: 0  Non exposed cohort representative: *  Exposure ascertainment: 0  Outcome not present at start: *  Controlled for weight: *  Controlled for age: *  Outcome assessment: *  Follow up duration: *  Follow up adequacy: * |
| Ness 2002 **UK ^2^** | DART trial follow up | 2033 men with coronary disease followed for 10.4 years | Cereal fibre intake obtained from FFQ | 1083 all-cause deaths, 739 due to CHD | History MI, angina, hypertension, cardiomegaly, pulmonary congestion, drugs | Exposed cohort representative: 0  Non exposed cohort representative: *  Exposure ascertainment: 0  Outcome not present at start: *  Controlled for weight: 0  Controlled for age: 0  Outcome assessment: *  Follow up duration: *  Follow up adequacy: * |
| Wu 2019 **Taiwan ^3^** | Biosignature study follow up | 716 adults with CAD followed for 2.2 years | Oat fibre intake obtained from interview | 12 deaths due to CVD | Age, gender, hypertension, diabetes, smoking, ratio of waist to hip, medication, lipid, HsCRP, TNF-α | Exposed cohort representative: 0  Non exposed cohort representative: *  Exposure ascertainment: 0  Outcome not present at start: *  Controlled for weight: 0  Controlled for age: *  Outcome assessment: *  Follow up duration: 0  Follow up adequacy: * |

**References**

1 Li S, Flint A, Pai JK, et al. Dietary fiber intake and mortality among survivors of myocardial infarction: prospective cohort study. BMJ 2014; 348.

2. Ness AR, Hughes J, Elwood PC, Whitley E, Smith G, Burr ML. The long-term effect of dietary advice in men with coronary disease: follow-up of the Diet and Reinfarction trial (DART). European Journal of Clinical Nutrition 2002; 56(6): 512-8.

3. Wu J-R, Leu H-B, Yin W-H, et al. The benefit of secondary prevention with oat fiber in reducing future cardiovascular event among CAD patients after coronary intervention. Scientific reports 2019; 9(1): 1-6.
