# Supplementary material for: Dietary fibre in hypertension and cardiovascular disease management: systematic review and meta-analyses
Source: BMC Med. 2022 Apr 22;20:139. doi: 10.1186/s12916-022-02328-x (PMC9027105; doi:10.1186/s12916-022-02328-x)
Supplement: Supplementary file 3 — Additional file 3: Table 1. Description of identified CVD trials. [file 12916_2022_2328_MOESM3_ESM.docx]

**Additional file 3 Table 1: Description of identified controlled trials of adults with cardiovascular disease reporting on fibre intake and cardiometabolic risk factors.**

| ID | Design | Participants | Daily intervention (I) | Daily control (C) | Outcomes | Duration | Fibre difference between I & C | Cochrane Risk of Bias tool |
| --- | --- | --- | --- | --- | --- | --- | --- | --- |
| **Trials of adults with CVD** | | | | | | | | |
| Salenius 1995  **Finland** | Randomised controlled parallel trial | N 40 (8 women) mean age 63, mean body weight 75.8kg. Participants with moderate hypercholesterolaemia recruited following carotid endarterectomy. No drug treatment for Hypercholesterolaemia. Body weight did not change (values not reported) | Guar gum | Placebo | Total Cholesterol  LDL  HDL  Triglycerides | 24 months | 15g | Sequence generation: U  Allocation concealment: U  Blinding of participants: L  Blinding of outcome: U  Incomplete data: L  Selective reporting: L  Other: U |
| Jang 2001  **Korea** | Randomised controlled parallel trial | N 76 (male) mean age 56, mean BMI 24.7 with Coronary Artery Disease | Replaced refined rice at breakfast with 70g whole grain and legume powder | Maintained refined rice intake | Total Cholesterol  LDL  HDL  Triglycerides  BMI  Fasting glucose  Fasting insulin  SBP  DBP | 16 weeks | 2.8g | Sequence generation: U  Allocation concealment: U  Blinding of participants: U  Blinding of outcome: U  Incomplete data: L  Selective reporting: L  Other: L |
| Vajifdar 2002  **India** | Randomised controlled parallel trial | 114 (23 women) mean age 56, mean BMI 24 with Coronary Artery Disease | 4.6g of fibre supplement consumed twice a day | placebo powder consumed twice a day | Total Cholesterol  LDL  HDL  Triglycerides  Body weight  BMI  WC  Fasting glucose | 6 months | 9.2g | Sequence generation : U  Allocation concealment: L  Blinding of participants: L  Blinding of outcome: L  Incomplete data: L  Selective reporting: L  Other : L |

**References**

1. Salenius J-P, Harju E, Jokela H, Riekkinen H, Silvasti M. Long term effects of guar gum on lipid metabolism after carotid endarterectomy. BMJ 1995; 310(6972): 95.

2. Jang Y, Lee JH, Kim OY, Park HY, Lee SY. Consumption of whole grain and legume powder reduces insulin demand, lipid peroxidation, and plasma homocysteine concentrations in patients with coronary artery disease: randomized controlled clinical trial. Arteriosclerosis, thrombosis, and vascular biology 2001; 21(12): 2065-71.

3. Vajifdar B, Goyal V, Lokhandwala Y, et al. Is dietary fiber beneficial in chronic ischemic heart disease? The Journal of the Association of Physicians of India 2000; 48(9): 871-6.
