# Supplementary material for: Dietary fibre in hypertension and cardiovascular disease management: systematic review and meta-analyses
Source: BMC Med. 2022 Apr 22;20:139. doi: 10.1186/s12916-022-02328-x (PMC9027105; doi:10.1186/s12916-022-02328-x)
Supplement: Supplementary file 4 — Additional file 4: Table 1. Description of identified hypertension trials. [file 12916_2022_2328_MOESM4_ESM.docx]

**Additional file 4 Table 1: Description of identified controlled trials of adults with hypertension reporting on fibre intake and cardiometabolic risk factors.**

| ID | Design | Participants | Daily intervention (I) | Daily control (C) | Outcomes | Duration | Fibre difference between I & C | Cochrane Risk of Bias tool |
| --- | --- | --- | --- | --- | --- | --- | --- | --- |
| **Trials of adults with hypertension** | | | | | | | | |
| Burke 2001  **Australia** | Randomised controlled parallel trial | N 18 (11 women) mean age 56.5 mean weight 79.5kg. Eligibility included on hypertensive agents and SBD 130-160 mmHg | 15g psyllium and 66g maltodextrin powder | 66g maltodextrin powder | SBP  DBP | 8 weeks | 12g | Sequence generation: L  Allocation concealment: U  Blinding of participants: H  Blinding of outcome: U  Incomplete data: L  Selective reporting: L  Other: L |
| Cicero 2007  **Italy** | Randomised controlled parallel trial with two fibre intervention arms and one control arm | N 141 (67 women) mean age 58 mean BMI 26.6. Eligibility included SBP >140 or DBP >90 mmHg, BMI 25-30, could include hypertensive agents | 7g of psyllium husk (48 participants) or 7g guar gum (48 other participants) | Standard diet (no placebo) | BMI  Total cholesterol  LDL  HDL  Triglycerides  SBP  DBP  Fasting insulin  Fasting glucose | 6 months | 5.6g | Sequence generation: L  Allocation concealment: L  Blinding of participants: L  Blinding of outcome: L  Incomplete data: L  Selective reporting: L  Other: L |
| Eliasson 1992  **Sweden** | Randomised controlled parallel trial | 63 (24 women) mean age 48 mean BMI 25.  Eligibility assessed as 2 supine DBP measures 90-115 mmHg. No current antihypertensive use | 7g fibre in tablet form | Placebo tablets containing 1g fibre | BMI  Total cholesterol  HDL  Triglycerides  SBP  DBP  Body weight  Fasting insulin  Fasting glucose  HbA1c | 3 months | 6g | Sequence generation: U  Allocation concealment: L  Blinding of participants: L  Blinding of outcome: L  Incomplete data: L  Selective reporting: L  Other: L |
| He 2004  **USA** | Randomised controlled parallel trial | N 110 (66 women)  mean age 48 mean BMI 29. Eligibility included SBP 125-159 and DBP <95 mmHg average from three screening visits.  No current antihypertensive use. | 60g oat bran and 84g oatmeal | 93g refined wheat and 42g corn flakes | Body weight  SBP  DBP | 12 weeks | 10.7g | Sequence generation: L  Allocation concealment: L  Blinding of participants: L  Blinding of outcome: L  Incomplete data: L  Selective reporting: L  Other: U |
| Maki 2007  **USA** | Randomised controlled parallel trial | Per protocol analysis of N 60 (27 women) mean age 59 mean BMI 32. Eligibility included SBP 130-179 and/or DBP 85-109 mmHg and waist circumference >87 for women or >95 cm for men. Included hypertensive use. | Oat products and oat beta-glucan powder | Low fibre cereals and maltodextrin powder | Fasting insulin  Fasting glucose | 12 weeks | 12g | Sequence generation: U  Allocation concealment: L  Blinding of participants: L  Blinding of outcome: L  Incomplete data: L  Selective reporting: H  Other: U |
| Pins 2002  **USA** | Randomised controlled parallel trial | N 88 (43 women) mean age 48 mean BMI 31. Eligibility included one  hypertensive use but with BP <160/100. Medications were assessed and changed throughout interventions. | 137g oat cereals | 146g low fibre cereals | Fasting glucose  Body weight  SBP of participants who did not reduce medication  DBP of participants who did not reduce medication  Total cholesterol  LDL  HDL  Triglycerides | 12 weeks | 8.1g | Sequence generation: U  Allocation concealment: L  Blinding of participants: L  Blinding of outcome: L  Incomplete data: L  Selective reporting: L  Other : H |
| Schlamowitz 1987 **Denmark** | Randomised controlled parallel trial | N 46 lean patients with a BP between 140/95 and 195/110 mmHg. | Fibre tables | Placebo tablets | SBP  DBP  Total cholesterol | 3 months | 7g | Sequence generation: U  Allocation concealment: U  Blinding of participants: U  Blinding of outcome: U  Incomplete data: H  Selective reporting: H  Other : H |
| Xue 2021  **China** | Randomised controlled parallel trial | N 50 (13 women) mean age 48 mean BMI 25. Eligibility criteria included SBP 140-159 and/or DBP 90-99 mmHg. | 30g oat bran | Not stated | SBP  DBP | 3 months | 8.3g | Sequence generation: L  Allocation concealment: U  Blinding of participants: H  Blinding of outcome: H  Incomplete data: H  Selective reporting: U  Other : H |
| Yoshinuma 2019 **Japan** | Randomised controlled parallel trial | N 72 mean BMI 24. Eligibility criteria included SBP 130-139 and/or DBO 85-89 mmHg, high dietary sodium intakes, and BMI 18-35 | Psyllium husk with 10.8g dietary fibre | Placebo powder with no fibre | SBP  DBP | 12 weeks | 10.8g | Sequence generation: U  Allocation concealment: U  Blinding of participants: L  Blinding of outcome: U  Incomplete data: H  Selective reporting: H  Other : U |

**References**

1. Burke V, Hodgson JM, Beilin LJ, Giangiulioi N, Rogers P, Puddey IB. Dietary protein and soluble fiber reduce ambulatory blood pressure in treated hypertensives. Hypertension 2001; 38(4): 821-6.

2. Cicero AF, Derosa G, Manca M, Bove M, Borghi C, Gaddi AV. Different effect of psyllium and guar dietary supplementation on blood pressure control in hypertensive overweight patients: a six-month, randomized clinical trial. Clinical and experimental hypertension 2007; 29(6): 383-94.

3. Eliasson K, Ryttig KR, Hylander B, Rössner S. A dietary fibre supplement in the treatment of mild hypertension. A randomized, double-blind, placebo-controlled trial. Journal of hypertension 1992; 10(2): 195-9.

4. He J, Streiffer RH, Muntner P, Krousel-Wood MA, Whelton PK. Effect of dietary fiber intake on blood pressure: a randomized, double-blind, placebo-controlled trial. Journal of hypertension 2004; 22(1): 73-80.

5. Maki K, Galant R, Samuel P, et al. Effects of consuming foods containing oat β-glucan on blood pressure, carbohydrate metabolism and biomarkers of oxidative stress in men and women with elevated blood pressure. European journal of clinical nutrition 2007; 61(6): 786-95.

6. Pins JJ, Geleva D, Keenan JM, Frazel C, O’Connor PJ, Cherney LM. Do whole-grain oat cereals reduce the need for antihypertensive medications and improve blood pressure control? The Journal of family practice 2002; 51(4): 353.

7. Schlamowitz P, Halberg T, Warnoe O, Wilstrup F, Ryttig K. Treatment of mild to moderate hypertension with dietary fibre. Lancet 1987.

8. Xue Y, Cui L, Qi J, et al. The effect of dietary fiber (oat bran) supplement on blood pressure in patients with essential hypertension: A randomized controlled trial. Nutrition, Metabolism and Cardiovascular Diseases 2021.

9. Yoshinuma H. Effects of Intake of Psyllium Husk on Blood Pressure in Subjects with High-normal Blood Pressure: Randomized, Double blind, Placebo controlled Parallel group Study. Japanese Pharmacology and Therapeutics 2019; 47(9): 1519-27.
