# Supplementary material for: Dietary fibre in hypertension and cardiovascular disease management: systematic review and meta-analyses
Source: BMC Med. 2022 Apr 22;20:139. doi: 10.1186/s12916-022-02328-x (PMC9027105; doi:10.1186/s12916-022-02328-x)
Supplement: Supplementary file 5 — Additional file 5: Analyses shown in full. Figure 1. Fibre and all-cause mortality meta analysis. Figure 2. Cereal fibre and all-cause mortality meta analysis. Figure 3. Fibre and CVD mortality meta analysis. Figure 4. Cereal fibre and CVD mortality meta analysis. Figure 5. Fibre and total cholesterol in hypertension meta analysis. Figure 6. Fibre and total cholesterol in hypertension dose controlled meta analysis. Figure 7. Fibre and HDL cholesterol in hypertension meta analysis. Figure 8. Fibre and HDL cholesterol in hypertension dose controlled meta analysis. Figure 9. Fibre and triglycerides in hypertension meta analysis. Figure 10. Fibre and triglycerides in hypertension dose controlled meta analysis. Figure 11. Fibre and fasting plasma glucose in hypertension meta analysis. Figure 12. Fibre and fasting plasma glucose in hypertension dose controlled meta analysis. Figure 13. Fibre and fasting plasma insulin in hypertension meta analysis. Figure 14. Fibre and fasting plasma insulin in hypertension dose controlled meta analysis. Figure 15. Fibre and systolic blood pressure in hypertension meta analysis. Figure 16. Fibre and systolic blood pressure in hypertension dose controlled meta analysis. Figure 17. Fibre and diastolic blood pressure in hypertension meta analysis. Figure 18. Fibre and diastolic blood pressure in hypertension dose controlled meta analysis. [file 12916_2022_2328_MOESM5_ESM.docx]

**Additional file** 5

**Observational study data:**

**Fibre and all-cause mortality**

**Figure 1**: Extreme quantile meta analyses of dietary fibre and all-cause mortality

**Figure 2**: Extreme quantile meta analyses of cereal fibre and all-cause mortality

Fibre and cardiovascular disease mortality

**Figure 3**: Extreme quantile meta analyses of dietary fibre and cardiovascular disease mortality

**Figure 4**: Extreme quantile meta analyses of cereal fibre and cardiovascular disease mortality

Egger p for bias 0573.

Influence analysis: The result from one study (DART trial) was observed to drive the pooled response. The point estimate of pooled sample without the DART trial was RR 0.73 (0.53 to 0.99) and the I^2^ was reduced to 0%.

**Trial data: sensitivity analyses for pooled data with four or more data points.**

**Fibre hypertension Total cholesterol**

**Figure 5**: Meta analyses of fibre interventions regardless of dose

Egger p for bias 0.797

Influence analysis: point estimate of pooled sample without either Eliasson 1992 or Pins 2002 was outside the confidence interval of the complete data set (one in each direction). High I2 most likely due to the precision around each trial’s observed effect size.

**Univariate**

Meta regression by trial duration (<or =12 vs >12). -.15 (-.91 to 0.61) p 0.579

Meta regression by antihypertensive use -0.30 (-1.73 to 1.14) p 0.469

Meta regression by food or supplements **-0.52 (-0.78 to -0.26) p 0.008 I2 reduced to 68%**

Meta regression by higher BMI -0.20 (-0.83 to 0.53) p 0.454

Meta regression by placebo control -0.15 (-0.91 to 0.61) p 0.579

Meta regression by psyllium 0.11 (-0.86 to 1.08) p 0.740

Insufficient data to consider dose response.

**Figure 6**: Meta analyses of fibre interventions with effect size standardised to 5g of fibre per day

**Fibre hypertension HDL cholesterol**

**Figure 7**: Meta analyses of fibre interventions regardless of dose

Egger p for bias 0.534

Influence analysis: point estimate of pooled sample without either Eliasson 1992 or Cicero 2007 guar gum intervention was outside the confidence interval of the complete data set (one in each direction).

**Univariate**

Meta regression by trial duration (<or =12 vs >12). 1.57 (-2.18 to 5.32) p 0.214

Meta regression by antihypertensive use -1.52 (-6.82 to 3.78) p 0.342

Meta regression by food or supplements 0.58 (-6.21 to 7.36) p 0.750

Meta regression by higher BMI -1.52 (-6.82 to 3.78) p 0.342

Meta regression by placebo control 1.57 (-2.18 to 5.32) p 0.214

Meta regression by psyllium 0.00 (-7.03 to 7.04) p0.999

Meta regression by geographic region (Europe or USA) -0.58 (-7.36 to 6.21) p 0.750

Insufficient data to consider dose response.

**Figure 8**: Meta analyses of fibre interventions with effect size standardised to 5g of fibre per day

**Fibre hypertension triglycerides**

**Figure 9**: Meta analyses of fibre interventions regardless of dose

Egger p for bias 0.752

Influence analysis: One study, Pins 2002 appreciably influenced the pooled result. Without Pins 2002 the pooled effect size estimate was -0.24 (-0.38 to -0.10).

**Univariate**

Meta regression by trial duration (<or =12 vs >12). -0.07 (-0.88 to 0.748) p 0.756

Meta regression by antihypertensive use 0.28 (-0.23 to 0.78) p 0.144

Meta regression by food or supplements 0.18 (-0.61 to 0.98) p 0.426

Meta regression by higher BMI 0.28 (-0.23 to 0.78) p 0.144

Meta regression by placebo control -0.07 (-0.88 to 0.748) p 0.756

Meta regression by psyllium -0.6 (-1.01 to 0.89) p 0.809

Meta regression by geographic region (Europe or USA) -0.18 (-0.98 to 0.61) p 0.426

Insufficient data to consider dose response.

**Figure 10**: Meta analyses of fibre interventions with effect size standardised to 5g of fibre per day

**Fibre hypertension fasting plasma glucose**

**Figure 11**: Meta analyses of fibre interventions regardless of dose

Egger p for bias 0.547

Influence analysis: point estimate of pooled sample without either Maki 2007 or Pins 2002 was outside the confidence interval of the complete data set (one in each direction).

**Univariate**

Meta regression by trial duration (<or =12 vs >12). 0.20 (-1.18 to 1.58) p 0.680

Meta regression by antihypertensive use -0.10 (-1.85 to 1.64) p 0.864

Meta regression by food or supplements 0.13 (-1.28 to 1.54) P 0.787

Meta regression by higher BMI -0.10 (-1.85 to 1.64) p 0.864

Meta regression by placebo control 0.20 (-1.18 to 1.58) p 0.680

Meta regression by psyllium -0.52 (-1.98 to 0.93) p 0.336

Meta regression by geographic region (Europe or USA) -0.13 (-1.54 to 1.28) p 0.787

Insufficient data to consider dose response.

**Figure 12**: Meta analyses of fibre interventions with effect size standardised to 5g of fibre per day

**Fibre hypertension fasting plasma insulin**

**Figure 13**: Meta analyses of fibre interventions regardless of dose

Egger p for bias 0.770

Influence analysis: point estimate of pooled sample without either Maki 2007 or Cicero 2002 was outside the confidence interval of the complete data set (one in each direction).

**Univariate**

Meta regression by trial duration (<or =12 vs >12). 3.4 (-18.2 to 25.0) p 0.568

Meta regression by antihypertensive use 3.6 (-21.3 to 28.6) p 0.594

Meta regression by food or supplements 9.3 (-3.3 to 22.0) p 0.087

Meta regression by higher BMI 3.6 (-21.3 to 28.6) p 0.594

Meta regression by placebo control 3. (-18.2 to 25.0) p 0.568

Meta regression by psyllium -3.8 (-28.2 to 20.6) p 0.572

Meta regression by geographic region (Europe or USA) -9.3 (-22.0 to 3.3) p 0.087

Insufficient data to consider dose response.

**Figure 14**: Meta analyses of fibre interventions with effect size standardised to 5g of fibre per day

**Fibre hypertension systolic blood pressure**

**Figure 15**: Meta analyses of fibre interventions regardless of dose

Egger p for bias 0.119

Influence analysis: One study, Eliasson 1992 appreciably influenced the pooled result. Without Eliasson 1992 the pooled effect size estimate was -4.7 (-6.3 to -3.1) I2 99%.

**Univariate**

Meta regression by trial duration (<or =12 vs >12). -3.0 (-10.0 to 3.9) p 0.337

Meta regression by antihypertensive use -3.0 (-9.6 to 3.7) p 0.306

Meta regression by food or supplements 0.6 (-6.9 to 8.2) p 0.851

Meta regression by higher BMI 1.6 (-4.9 to 8.1) p 0.583

Meta regression by placebo control 0.3 (-6.3 to 7.0) p 0.915

Meta regression by psyllium 2.4 (-3.9 to 8.8) p 0.396

Meta regression by geographic region (Europe or other) 0.5 (-5.8 to 6.8) p 0.852

Meta regression by geographic region (Asia or other) -2.2 (-9.5 to 5.1) p 0.499

Meta regression by geographic region (North America or other) 0.6 (-6.9 to 8.2) p 0.851

**Figure 16**: Meta analyses of fibre interventions with effect size standardised to 5g of fibre per day

**Fibre hypertension diastolic blood pressure**

**Figure 17**: Meta analyses of fibre interventions regardless of dose

Egger p for bias 0.859

Influence analysis: One study, Yoshinuma 2019 appreciably influenced the pooled result. Without Yoshinuma 2019 the pooled effect size estimate was -3.4 (-4.9 to -1.9) I2 99%.

**Univariate**

Meta regression by trial duration (<or =12 vs >12). -2.8 (-8.5 to 2.9) p 0.281

Meta regression by antihypertensive use -0.2 (-8.2 to 7.7) p 0.941

Meta regression by food or supplements -0.0 (-6.3 to 6.2) p 0.990

Meta regression by higher BMI 1.2 (-4.2 to 6.6) p 0.608

Meta regression by placebo control 1.1 (-4.3 to 6.5) p 0.649

Meta regression by psyllium 3.3 (-1.5 to 8.0) p 0.148

Meta regression by geographic region (Europe or other) 1.0 (-4.2 to 6.1) p 0.666

Meta regression by geographic region (Asia or other) -2.6 (-8.4 to 3.2) p 0.323

Meta regression by geographic region (North America or other) -0.0 (-6.2 to 6.2) p 0.990

**Figure 18**: Meta analyses of fibre interventions with effect size standardised to 5g of fibre per day
