# Supplementary material for: Dietary fibre in hypertension and cardiovascular disease management: systematic review and meta-analyses
Source: BMC Med. 2022 Apr 22;20:139. doi: 10.1186/s12916-022-02328-x (PMC9027105; doi:10.1186/s12916-022-02328-x)
Supplement: Supplementary file 6 — Additional file 6. GRADE tables of certainty of evidence. Table 1. GRADE table of fibre and mortality. Table 2. GRADE table of fibre in CVD management. Table 3. GRADE table of fibre in hypertension management. [file 12916_2022_2328_MOESM6_ESM.docx]

**Additional file 6: GRADE tables**

**Table 1: What is the role of higher fibre intakes when compared with low fibre intakes in mortality outcomes for adults with cardiovascular disease?**

| **Certainty assessment** | | | | | | | **№ of patients** | **Effect** | | **Certainty** |  |
| --- | --- | --- | --- | --- | --- | --- | --- | --- | --- | --- | --- |
| **№ of studies** | **Study design** | **Risk of bias** | **Inconsistency** | **Indirectness** | **Imprecision** | **Other considerations** | **Cases/cohort** | **Relative (95% CI)** | **Absolute (95% CI)** |  |  |
| **Dietary fibre and all-cause mortality** | | | | | | | | | | |  |
| 2 | observational studies | not serious | not serious | not serious | not serious | dose response gradient | 1133/4720 (24.0%) | **RR 0.75** (0.58 to 0.97) | **60 fewer per 1,000** (from 101 fewer to 7 fewer) | ⨁⨁⨁ MODERATE |  |
| **Cereal fibre and all-cause mortality** | | | | | | | | | | | |
| 3 | observational studies | not serious | serious ^a^ | not serious | serious ^b^ | none | 2216/6753 (32.8%) | **RR 0.90** (0.62 to 1.30) | **33 fewer per 1,000** (from 125 fewer to 98 more) | ⨁ VERY LOW |  |
| **Dietary fibre and CVD mortality** | | | | | | | | | | | |
| 2 | observational studies | not serious | not serious | not serious | serious ^b^ | none | 558/4720 (11.8%) | **RR 0.86** (0.60 to 1.24) | **17 fewer per 1,000** (from 47 fewer to 28 more) | ⨁ VERY LOW |  |
| **Cereal fibre and CVD mortality** | | | | | | | | | | | |
| 4 | observational studies | not serious | serious ^c^ | not serious | serious ^b^ | none | 1309/7469 (17.5%) | **RR 0.91** (0.64 to 1.31) | **16 fewer per 1,000** (from 63 fewer to 54 more) | ⨁ VERY LOW |  |

#### Explanations

a. Initial I^2^ was high (88.5%) with studies providing a significant risk reduction or a non significant risk increase. Evidence for this exposure and outcome downgraded once due to Inconsistency.

b. Confidence interval around the pooled point estimate contains both a strong beneficial effect (<0.8) and a strong detrimental effect (>1.2). Evidence for this exposure and outcome downgraded once due to Imprecision

c. Initial I^2^ was high (65.9%) with studies providing a significant risk reduction, a non significant risk reduction, and a non significant risk increase. Evidence for this exposure and outcome downgraded once due to Inconsistency.

**Table 2: What is the role of increasing fibre intakes on cardiometabolic risk factors in the management of cardiovascular disease?**

| **Certainty assessment** | | | | | | | **№ of patients** | | **Effect** | **Certainty** |
| --- | --- | --- | --- | --- | --- | --- | --- | --- | --- | --- |
| **№ of studies** | **Study design** | **Risk of bias** | **Inconsistency** | **Indirectness** | **Imprecision** | **Other considerations** | **Intervention** | **Control** | **Absolute (95% CI)** |  |
| **Total cholesterol** | | | | | | | | | | |
| 3 | randomised trials | not serious | serious ^a^ | not serious | serious ^b^ | none | 117 | 110 | MD **0.42 mmol/L lower** (0.78 lower to 0.05 lower) | ⨁⨁ LOW |
| **LDL cholesterol** | | | | | | | | | | |
| 3 | randomised trials | not serious | serious ^a^ | not serious | serious ^b^ | none | 117 | 110 | MD **0.47 mmol/L lower** (0.85 lower to 0.1 lower) | ⨁⨁ LOW |
| **HDL cholesterol** | | | | | | | | | | |
| 3 | randomised trials | not serious | serious ^a^ | not serious | serious ^b^ | none | 117 | 110 | MD **0.08 mmol/L higher** (0.02 lower to 0.17 higher) | ⨁⨁ LOW |
| **Triglycerides** | | | | | | | | | | |
| 3 | randomised trials | not serious | serious ^a^ | not serious | serious ^b^ | none | 117 | 110 | MD **0.03 mmol/L lower** (0.15 lower to 0.08 higher) | ⨁⨁ LOW |
| **Systolic blood pressure** | | | | | | | | | | |
| 1 | randomised trials | not serious | very serious ^c^ | not serious | serious ^b^ | none | 38 | 38 | MD **1.2 mm Hg lower** (2 lower to 0.4 lower) | ⨁ VERY LOW |
| **Diastolic blood pressure** | | | | | | | | | | |
| 1 | randomised trials | not serious | very serious ^c^ | not serious | serious ^b^ | none | 38 | 38 | MD **3.6 mm Hg lower** (4 lower to 3.2 lower) | ⨁ VERY LOW |
| **Body weight** | | | | | | | | | | |
| 1 | randomised trials | not serious | very serious ^c^ | not serious | serious ^b^ | none | 61 | 53 | MD **0.2 kg lower** (0.37 lower to 0.04 lower) | ⨁ VERY LOW |
| **BMI** | | | | | | | | | | |
| 2 | randomised trials | not serious | serious ^a^ | not serious | serious ^b^ | none | 99 | 91 | MD **0.3 kg/m2 lower** (0.69 lower to 0.09 higher) | ⨁⨁ LOW |
| **Waist circumference** | | | | | | | | | | |
| 1 | randomised trials | not serious | very serious ^c^ | not serious | serious ^b^ | none | 61 | 53 | MD **0.5 cm lower** (0.6 lower to 0.4 lower) | ⨁ VERY LOW |
| **Fasting plasma glucose** | | | | | | | | | | |
| 2 | randomised trials | not serious | serious ^a^ | not serious | serious ^b^ | none | 99 | 91 | MD **1.23 mmol/L lower** (2.13 lower to 0.33 lower) | ⨁⨁ LOW |
| **Fasting plasma insulin** | | | | | | | | | | |
| 1 | randomised trials | not serious | very serious ^c^ | not serious | serious ^b^ | none | 38 | 38 | MD **10.8 pmol/L lower** (13.2 lower to 8.4 lower) | ⨁ VERY LOW |

#### Explanations

a. High initial heterogeneity, downgraded once for Inconsistency.

b. Insufficient data to provide comment on precision. Participant number in analyses were <300, unlikely to meet optimal information size parameters. Downgraded once.

c. With data from only one study Inconsistency cannot be assessed. Downgraded twice.

**Table 3: What is the role of increasing fibre intakes on cardiometabolic risk factors in the management of hypertension?**

| **Certainty assessment** | | | | | | | **№ of patients** | | **Effect** | **Certainty** |
| --- | --- | --- | --- | --- | --- | --- | --- | --- | --- | --- |
| **№ of studies** | **Study design** | **Risk of bias** | **Inconsistency** | **Indirectness** | **Imprecision** | **Other considerations** | **Intervention** | **Control** | **Absolute (95% CI)** |  |
| **Total cholesterol** | | | | | | | | | | |
| 5 | randomised trials | not serious | serious ^a^ | not serious | serious ^b^ | none | 190 | 144 | MD **22 mmol/L lower** (0.45 lower to 0.01 higher) | ⨁⨁ LOW |
| **LDL cholesterol** | | | | | | | | | | |
| 3 | randomised trials | not serious | serious ^a^ | not serious | serious ^c^ | none | 137 | 88 | MD **0.29 mmol/L lower** (0.4 lower to 0.17 lower) | ⨁⨁ LOW |
| **HDL cholesterol** | | | | | | | | | | |
| 4 | randomised trials | not serious | serious ^a^ | not serious | serious ^c^ | none | 169 | 119 | MD **0.02 mmol/L higher** (0.01 lower to 0.05 higher) | ⨁⨁ LOW |
| **Triglycerides** | | | | | | | | | | |
| 4 | randomised trials | not serious | serious ^a^ | not serious | serious ^c^ | none | 169 | 119 | MD **0.19 mmol/L lower** (0.3 lower to 0.08 lower) | ⨁⨁ LOW |
| **Systolic blood pressure** | | | | | | | | | | |
| 9 | randomised trials | not serious | not serious | not serious | not serious | none | 281 | 250 | MD **4.3 mm Hg lower** (5.8 lower to 2.8 lower) | ⨁⨁⨁⨁ HIGH |
| **Diastolic blood pressure** | | | | | | | | | | |
| 9 | randomised trials | not serious | not serious | not serious | not serious | none | 281 | 250 | MD **3.1 mm Hg lower** (4.4 lower to 1.7 lower) | ⨁⨁⨁⨁ HIGH |
| **Body weight** | | | | | | | | | | |
| 3 | randomised trials | not serious | serious ^a^ | not serious | serious ^c^ | none | 137 | 88 | MD **0.14 kg lower** (1.36 lower to 1.08 higher) | ⨁⨁ LOW |
| **BMI** | | | | | | | | | | |
| 2 | randomised trials | not serious | serious ^a^ | not serious | serious ^c^ | none | 92 | 45 | MD **1.3 kg/m2 lower** (2.1 lower to 0.5 lower) | ⨁⨁ LOW |
| **HbA1c** | | | | | | | | | | |
| **1** | randomised trials | not serious | very serious ^d^ | not serious | serious ^c^ | none | 32 | 31 | MD **0.3 % higher** (0.2 higher to 0.4 higher) | ⨁ VERY LOW |
| **Fasting plasma glucose** | | | | | | | | | | |
| 5 | randomised trials | not serious | serious ^a^ | not serious | not serious | none | 195 | 153 | MD **0.48 mmol/L lower** (0.91 lower to 0.05 lower) | ⨁⨁⨁ MODERATE |
| **Fasting plasma insulin** | | | | | | | | | | |
| 4 | randomised trials | not serious | serious ^a^ | not serious | serious ^c^ | none | 150 | 110 | MD **3.5 pmol/L lower** (5.5 lower to 1.6 lower) | ⨁⨁ LOW |

#### Explanations

a. High initial heterogeneity, downgraded once for Inconsistency.

b. The confidence interval spans both a strong beneficial effect and null effect. Downgraded once for imprecision.

c. Insufficient data to provide comment on precision. Participant number in analyses were <300, unlikely to meet optimal information size parameters. Downgraded once.

d. With data from only one study Inconsistency cannot be assessed. Downgraded twice.
